# Differential Pathophysiological Drivers of Susceptibility to Type 2 Diabetes and Metabolic Dysfunction–Associated Steatotic Liver Disease: Ethnic Differences in Insulin Dynamics, Whole‐Body Fat Metabolism, and Organ‐Specific Lipid Deposition

**DOI:** 10.1111/obr.70104

**Published:** 2026-02-16

**Authors:** Daniel J. Cuthbertson, Martin Whyte, Alex E. Henney, Uazman Alam, Louise Goff, Barbara A. Fielding, A. Margot Umpleby

**Affiliations:** ^1^ Department of Cardiovascular and Metabolic Medicine, Institute of Life Course and Medical Sciences University of Liverpool Liverpool UK; ^2^ University Hospital Aintree Liverpool University Hospitals NHS Foundation Trust Liverpool UK; ^3^ Department of Nutrition, Food and Exercise Sciences, Faculty of Health and Medical Sciences Guildford UK; ^4^ King's College Hospital London UK; ^5^ King's College London London UK; ^6^ Leicester Diabetes Centre Leicester General Hospital and University of Leicester Leicester UK

**Keywords:** diabetes, insulin resistance, metabolic dysfunction–associated steatotic liver disease, obesity

## Abstract

**Introduction:**

This narrative review explores the epidemiological evidence and potential underlying pathophysiological defects underlying the disproportionately greater risk of Type 2 diabetes (T2D) and cardiometabolic disease in people of South Asian and African Caribbean ancestry compared with White Europeans. Differences in (i) insulin dynamics, (ii) body composition and liver and pancreas triglyceride accumulation, and (iii) dysregulated fat metabolism likely contribute to this obesity‐related susceptibility.

**Insulin Dynamics:**

Insulin resistance and hyperinsulinemia are key pathophysiological defects in T2D, although the primary defect is uncertain. Many believe that insulin resistance precedes compensatory hyperinsulinemia; much data suggest that hyperinsulinemia precedes insulin resistance. Hyperinsulinemia, related to reduced hepatic insulin clearance, may represent the primary defect in people of African Caribbean ancestry.

**Body Composition:**

Ectopic fat, particularly visceral, liver, and pancreatic fat, is associated with impairments in insulin action/secretion: Higher liver fat is specifically related to hepatic insulin resistance and higher pancreatic fat to impaired beta cell function. People of South Asian ancestry exhibit greater ectopic particularly liver fat, compared with White Europeans, and more severe insulin resistance, driving hyperinsulinemia. People of African Caribbean ancestry have lower visceral and liver fat and greater muscle mass.

**Dysregulated Fat Metabolism:**

Dysregulated fat metabolism in adipose tissue/liver may increase serum fatty acids and triglyceride concentrations exposing non‐adipose tissues to increased lipid. Differential T2D susceptibility likely reflects diverse but ethnic group–specific metabolic phenotypes representing genetic and environmentally mediated pathophysiological traits, consistent with the “palette” model of T2D.

## 1 Introduction

We will consider first the epidemiological evidence underlying the differential susceptibility to Type 2 diabetes (T2D) and metabolic dysfunction–associated steatotic liver disease (MASLD) observed in individuals of South Asian and African Caribbean ancestry, compared with White Europeans, with T2D occurring at lower relative adiposity and associated with earlier, more aggressive diabetes‐related complications, before considering potential underlying pathophysiological defects. We shall consider only a comparison of individuals of South Asian, African Caribbean, and White European ancestry; other ethnic groups, for example, Hispanic and East Asian populations, living globally with an elevated T2D risk are not discussed.

### 1.1 Pathophysiology of Obesity‐Related Complications

Obesity is a complex chronic disease, related to abnormal adipose tissue mass, distribution, and function [1]. Caloric consumption, in excess of metabolic need, leads to energy being stored as fat in white adipose tissue (WAT). WAT expands by either hypertrophy or hyperplasia [2], with differences in propensity to these processes, dependent upon the anatomical site. Subcutaneous WAT can undergo some degree of hyperplasia, with new fat cells being generated from precursor cells. However, once maximal potential recruitment of pre‐adipocytes has been achieved, additional fat “spills over” into other tissues not designed for fat storage, including the liver, pancreas, skeletal muscle, and the heart. Obesity‐related complications occur due to this limited capacity of subcutaneous fat to store excess energy, and specifically, it is the accumulation of ectopic fat (i.e., fat distribution in tissues/organs not designed for fat storage) that drives the associated metabolic abnormalities (*lipotoxicity*) that are key in the pathophysiology of cardiometabolic diseases such as T2D and MASLD [3]. Hence, living with overweight and obesity represents principal contributor to development of T2D and MASLD.

### 1.2 Prevalence of Overweight and Obesity in Different Ethnic Groups

The prevalence of overweight and obesity (body mass index [BMI] ≥ 25 and ≥ 30 kg/m^2^, respectively) in the United Kingdom has increased dramatically in both men and women (67% and 60% of men/women living with overweight/obesity; 26% and 29% women living with overweight/obesity) [4]. Obesity, and its consequences, particularly T2D and MASLD, disproportionately affects ethnic minority groups in this country (particularly people of South/East Asian and of Black African and Caribbean ancestry). Rates of overweight/obesity are highest in Black people, affecting 72% of adults from Black ethnic groups [5]. These stark findings illustrate the health inequalities that exist not only between ethnic minority and White groups but also between different ethnic minority groups [6]. According to 2021 Census data, the population of England and Wales is 59.6 million, of which 81.7% (48.7 million) are White, Asian people (predominantly South Asian) comprise the second largest population percentage (9.3%; 5.5 million), and Black people (predominantly African Caribbean) comprise the third largest (4.0%; 2.4 million).

### 1.3 Interaction of Sex on T2D Risk

Across a range of ethnicities, men have a higher prevalence of T2D compared with age‐ and BMI‐matched women [7], with men diagnosed with T2D at a lower BMI than women (31.8 vs. 33.7 kg/m^2^) despite broadly similar levels of HbA1c. Mean BMI difference at diagnosis was most marked at a younger age and narrowed with advancing age [8]. This sex difference, relating to greater underlying insulin resistance and other features of the metabolic syndrome in men [9], is apparent in White people (men 6% vs. women 3.6%), South Asians (21 vs. 13.8%), and Black Africans (13.3 vs. 9.7%) [7]. Across these three ethnicities, women, particularly younger women, appear to undergo larger excess weight gain than men prior to being diagnosed with T2D [10].

### 1.4 Ethnic‐Specific Differences in T2D Risk

T2D is a complex disease with susceptibility influenced by genetic, environmental, and personal behavioral risk factors [11]. Although T2D prevalence varies widely according to sex, age, geographical region, and socioeconomic position, its prevalence is also significantly influenced by race and ethnicity. “Race” indicates continent or region of ancestral origin, whereas “ethnicity” indicates cultural identity. In people of South Asian or African Caribbean descent, living in the Western world, T2D is three to five times more prevalent compared to White Europeans. In a UK Biobank cohort of 500,000 participants, T2D prevalence was 18% in South Asians, 13% in African Caribbean, and 4% in Europeans [12].

**FIGURE 1:**
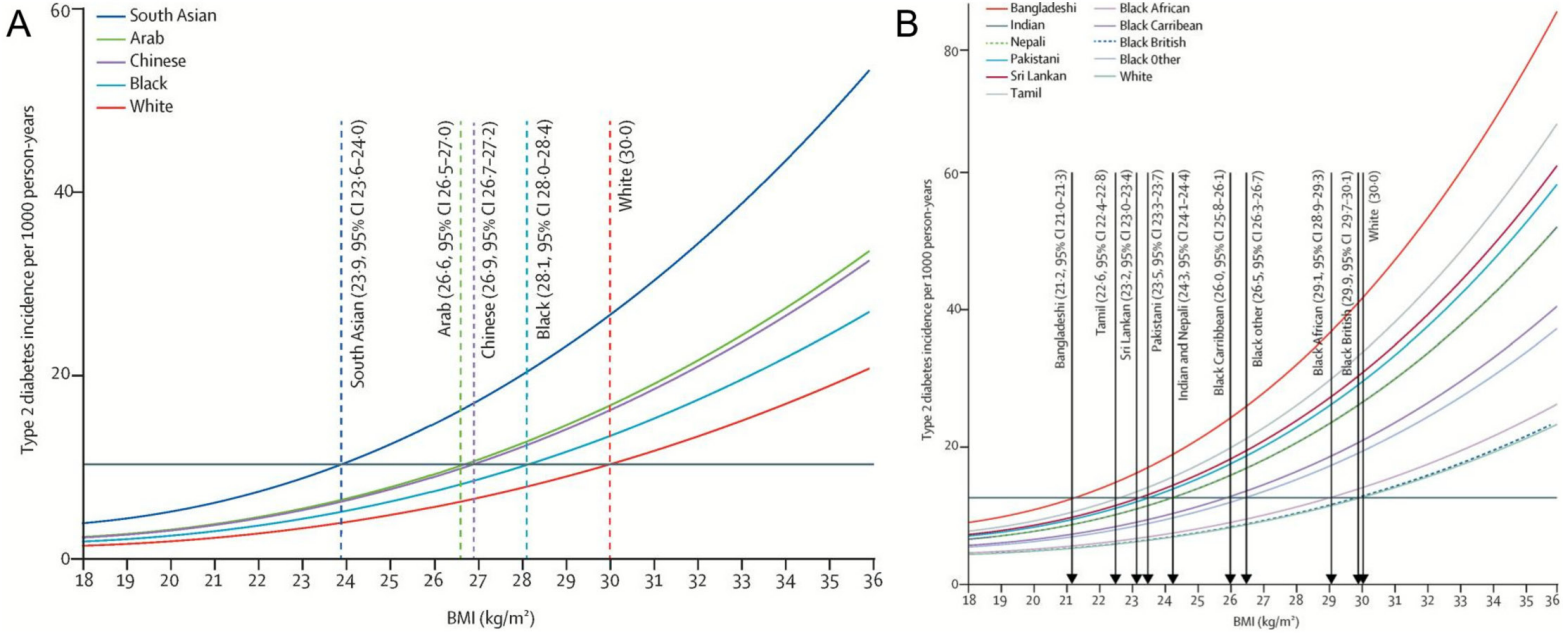
Age‐adjusted and sex‐adjusted BMI cutoffs equivalent to a BMI cutoff of 30.0 kg/m^2^ in White populations in relation to T2D incidence in England in (A) minority ethnic populations and (B) minority ethnic subgroups. The incidence of T2D for a BMI of 30.0 kg/m^2^ in the White population can be read off the graph at the intersection of the gray horizontal line and the fitted line for the White population. Taken from [14].

There is compelling evidence that minority ethnic groups are more sensitive to the effects of excess weight than White Europeans [12]. UK Biobank data demonstrate that T2D develops at a lower BMI in minority ethnic groups than in White people; for the same prevalence of T2D as BMI 30 kg/m^2^ in White people, BMI is 22 and 26 kg/m^2^ in South Asians and in Black people, respectively [13]. Similarly, in the Clinical Practice Research Datalink, an equivalent age‐ and sex‐adjusted incidence of T2D at a BMI of 30 kg/m^2^ in White Europeans was seen with BMI cutoffs of 23.9 kg/m^2^ in South Asian and 28.1 kg/m^2^ in Black populations [14] (Figure 1). This risk is lower in second‐generation (vs. first‐generation) migrants [12]. Ethnic‐specific BMI cutoffs of 23 and 27.5 kg/m^2^ have been proposed for detecting overweight and obesity, respectively, in South Asian populations [15], but there are currently no specific cutoffs for African Caribbean populations.

There are significant socioeconomic and environmental drivers of the elevated T2D risk in different ethnic groups, which can be explored by examining the prevalence of diabetes in migrants compared with that in the country of origin. Although the markedly elevated T2D risk in non‐White ethnic groups persists on migration to low‐risk countries, second‐generation migrants have a 20% lower risk compared with first generation [12]. It is unclear whether the inverse also holds true, where migration to higher‐risk countries could further amplify the risk of T2D. There is a significant interaction with socioeconomic deprivation [16], poorer diet, lower levels of physical activity, and reduced access to healthcare. It is not clear whether and/or to what extent each of these contributes to ethnic susceptibility although genetic ancestry associations are likely to be confounded by environmental and behavioral risk factors. Several interesting observations provide mechanistic insight into the ethnic‐specific risk. These may be considered to be related to differences in body composition and in organ‐specific (liver and pancreas) triacylglycerol (triglycerides [TG]) accumulation and related to this dysregulation of both insulin dynamics and fat metabolism. The following sections summarize the evidence for each pathophysiological driver.

## 2 Differences in Body Composition According to Ethnicity

The impact of different body composition phenotypes has been elegantly shown in recent genetic studies utilizing multiorgan imaging and longer‐term disease outcomes. They have been discussed in the context of ethnicity [17]. Such studies have identified common genetic variants (**favorable adiposity alleles**) with divergent effects on adiposity and risk of cardiometabolic disease: higher body fat percentage and higher BMI but lower risk of T2D, cardiovascular disease (CVD), and hypertension, attributed to greater subcutaneous storage capacity [18, 19]. Subsequent studies incorporating GWAS data and MRI imaging assessments confirm this healthier phenotype as being associated with greater SAT volume and less ectopic fat including VAT and liver fat [20, 21]. These genetic variants, associated with favorable and unfavorable adiposity, have consistent effects on metabolic profiles and disease risk across diverse ethnic groups including **European, Africa**n, and Central/South Asian [22].

Regional fat distribution (subcutaneous adipose tissue [SAT] vs. visceral adipose tissue [VAT]), organ‐specific fat accumulation, and lean body (skeletal muscle) mass differ between ethnicities.

### 2.1 SAT and VAT

Regarding SAT and VAT, a recent systematic review and meta‐analysis suggested that South Asian men had more SAT and liver fat than White European counterparts but similar amounts of VAT despite a lower BMI [21]. South Asian women also had a lower BMI and higher liver fat compared with White European women and similar amounts of VAT, but unlike the men, there was no difference in SAT. However, there are clearly also ethnic‐specific differences in distinct sub‐compartments of SAT: superficial versus deep (SSAT vs. DSAT) [23], which is a largely undeveloped research area.

### 2.2 Intrahepatic and Intrapancreatic Fat

Higher intrahepatic lipid is noted in South Asian people [21, 24, 25, 26] in some, although not all, studies (involving where possible age‐ and BMI‐matched individuals), whereas a recent systematic review/meta‐analysis noted lower intrahepatic lipid in Black African men compared with other ethnicities [27]. Pancreatic fat has also been shown (in the same systematic review/meta‐analysis) to differ by ethnicity, potentially affecting insulin secretion [27]. Studies comparing White Europeans with Black Africans found lower pancreatic fat in Africans [27]. These differences in adipose tissue versus ectopic fat distribution across the three ethnic groups provide a basis for their differential risk of developing T2D, MASLD, and CVD [14].

### 2.3 Lean Body Mass

Similarly, there are differences in lean body mass between ethnic groups: lowest in South Asians and highest in Black people [28].

In summary, adverse body composition changes with higher levels of ectopic fat deposition and greater peripheral (particularly gluteo‐femoral) fat deposition in South Asians compared to Whites seem to explain the development of peripheral and hepatic insulin resistance. In contrast, as Black Africans have lower levels of ectopic fat, we must consider that either Black Africans are more sensitive to ectopic fat deposition or ectopic fat is not a significant mediator of T2D within this population [29].

## 3 Ethnic Differences in Insulin Dynamics

### 3.1 Insulin Dynamics and Regulation of Glucose Metabolism Under Normal Conditions

Normal glucose regulation is dependent upon adequate insulin secretion, optimal target tissue insulin sensitivity, and insulin clearance from the systemic circulation; these various physiological processes can be considered under the umbrella term, insulin dynamics.

#### 3.1.1 Normal Secretion and Insulin Clearance

**FIGURE 2:**
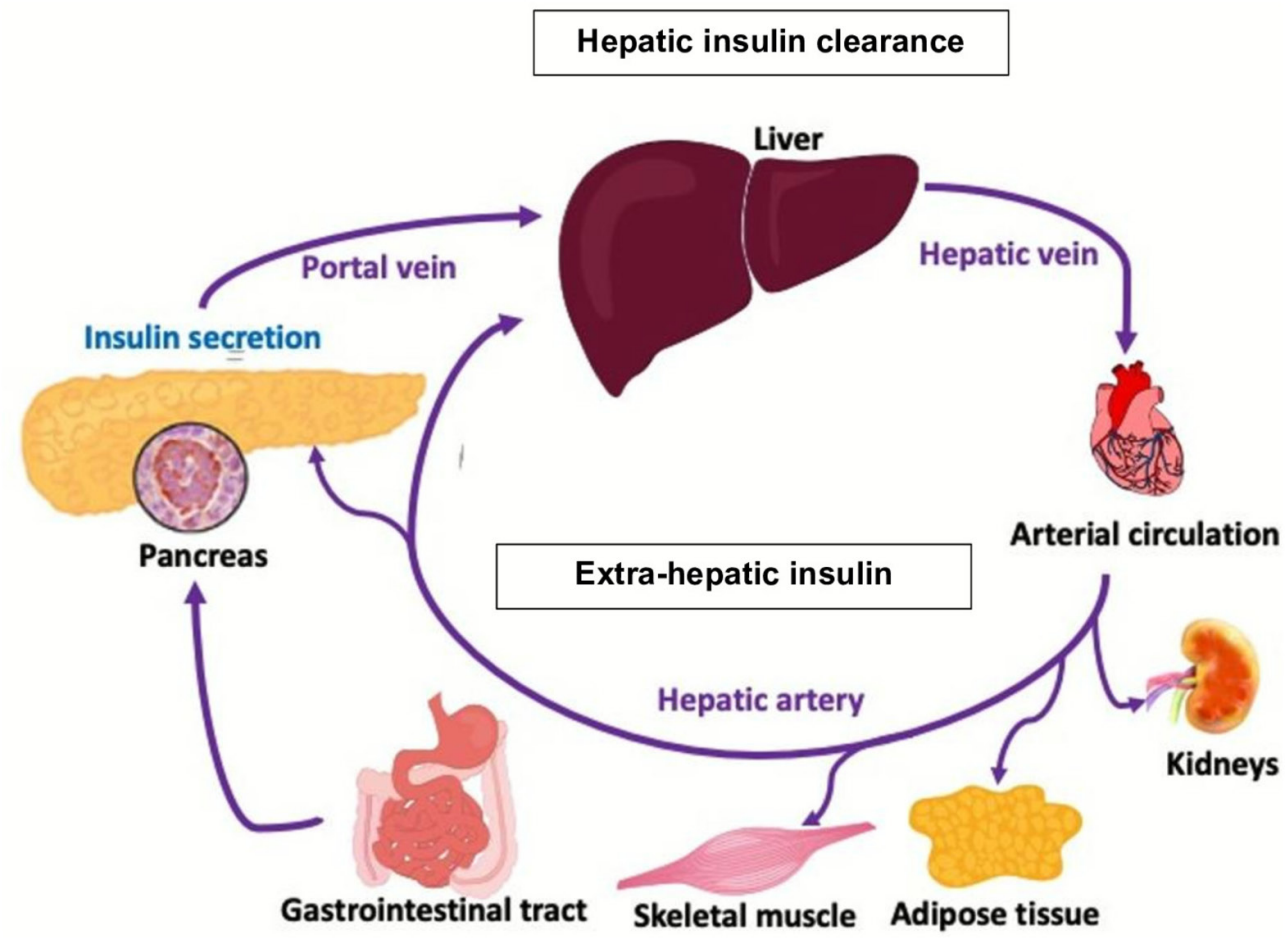
Insulin clearance: The liver is the first organ to be exposed to the secreted insulin via the portal vein. During its first pass through the liver, there is receptor‐mediated insulin uptake with half of the insulin secreted being degraded (*hepatic insulin clearance*), with the other half reaching the systemic circulation, with some degradation in other tissues such as skeletal muscle and adipocytes (*extrahepatic insulin clearance*), and with the remaining insulin returning to the liver for degradation and eventually being terminally degraded in the renal proximal tubular cells.

Pancreatic beta cells release insulin into the portal circulation, under fasted conditions, in a pulsatile fashion with secretory bursts every 5–15 min [30]. The liver is the first organ to be exposed to the secreted insulin via the portal vein. Within the liver, insulin passes through fenestrae (pores) in hepatic sinusoids and binds to hepatocyte membrane‐bound receptors. After insulin action, the insulin–insulin receptor complex is directed to lysosomal compartments within the hepatocyte where insulin undergoes degradation and the insulin receptor may undergo either lysosomal degradation or recycle back to the plasma membrane [31]. This process of receptor‐mediated insulin uptake and degradation serves as the fundamental mechanism for insulin clearance. Approximately half of the insulin secreted is degraded, in this manner, during its first pass through the liver (*hepatic insulin clearance*), with the other half reaching the systemic circulation, with some degradation in other tissues such as skeletal muscle and adipocytes (*extrahepatic insulin clearance*), and with the remaining insulin returning to the liver for degradation and eventually being terminally degraded in the renal proximal tubular cells [32] (Figure 2).

There is wide variation among individuals in the fraction of insulin cleared by the liver (20%–80%): the metabolic clearance rate (MCR). This variable is critically important as the rates of hepatic insulin clearance will also influence peripheral insulin concentrations. Circulating plasma insulin concentration is determined not only by pancreatic beta cell insulin secretion but also by (hepatic/extra‐hepatic) insulin clearance. Lower hepatic insulin clearance, for example, would result in peripheral hyperinsulinemia, which may exacerbate insulin resistance. A number of factors are associated with a reduced MCR of insulin including obesity [33], T2D and ethnicity, with a lower MCR in Black African people. Using insulin and C‐peptide measurements, first‐pass hepatic insulin extraction was demonstrated to be approximately two‐thirds lower in African Caribbean volunteers compared with their European counterparts [34]. Similar differences were also found in children of African Caribbean and White origin [35, 36]. However, in each case, there were no differences in extrahepatic insulin extraction. Significantly, lower MCR of insulin is prospectively associated with an increased risk of T2D [37].

#### 3.1.2 Ectopic Fat and Development of Insulin Resistance

The traditional paradigm of T2D pathophysiology is that adiposity generates insulin resistance, leading to hyperinsulinemia and eventual beta cell failure/deficient insulin secretion (Figure S1).

In contrast to subcutaneous fat, intra‐abdominal (visceral) fat expands primarily through hypertrophy exposing the hypertrophied adipocytes to oxygen deprivation, due to the greater cellular distance to traverse from the exiting microcirculation. Subsequent hypoxia activates the pro‐inflammatory mediator, hypoxia‐inducible factor (HIF), upregulating secretion of pro‐inflammatory cytokines (such as IL‐6 and TNF‐α) and activating immune cells [38]. The inflammatory cytokines, through impairment of insulin signal transduction pathways in the liver, skeletal muscle, and adipose tissue, induce peripheral and hepatic insulin resistance [39]. This pro‐inflammatory effect of VAT is compounded by the venous drainage of VAT into the portal circulation (vs. the systemic circulation from SAT). Increased free fatty acid flux is associated with increased hepatic TG synthesis and storage and further impairment of hepatic insulin sensitivity. This may be exacerbated by the particularly high turnover of VAT, compared to other fat depots [40].

#### 3.1.3 Insulin Resistance and Hyperinsulinemia

To overcome insulin resistance, there is a compensatory increase in pancreatic insulin secretion and hyperinsulinemia. Insulin resistance is also associated with a loss of insulin pulsatility, which will further compromise insulin signalling, endocytosis, and degradation, perpetuating chronic hyperinsulinemia [41]. A consequence of chronic hyperinsulinemia is activation of the transcription factor, sterol regulatory element‐binding protein‐1c (SREBP‐1c), which promotes expression of fatty acid synthase (FASN), a critical enzyme involved in fatty acid synthesis, such that increased lipogenesis occurs. Liver fat accumulation further impairs insulin clearance and insulin resistance and ultimately ever greater hyperinsulinemia. At some stage, when hyperinsulinemia is unable to overcome the degree of insulin resistance, T2D ensues.

This “traditional” paradigm of T2D pathophysiology with primary insulin resistance and secondary hyperinsulinemia was predominantly derived from the findings in studies undertaken in White European people. The prevailing view is that a similar pathophysiology is relevant for T2D in South Asian people. In support of this model, increased insulin resistance is present for several years before the diagnosis of prediabetes or T2D in South Asians [42, 43, 44], driven by both increased ectopic fat and reduced insulin‐mediated glucose uptake due to less lean mass (skeletal muscle mediates ~80% of insulin‐mediated glucose uptake) [45, 46, 47, 48]. However, the “traditional” paradigm, as outlined above, has been challenged as being primarily responsible for T2D in South Asians, and it may be that an alternative explanation is that impaired insulin secretion may be of greater importance [49, 50, 51, 52] (Figure S2).

#### 3.1.4 Reduced Beta Cell Function in South Asians

Cross‐sectional analysis of the Mediators of Atherosclerosis in South Asians Living in America (MASALA) study and the Multi‐Ethnic Study of Atherosclerosis (MESA) showed that South Asians had the lowest insulin secretion of all ethnic groups studied. There are few data on hepatic insulin clearance in South Asians [44]. In a Norwegian study, South Asian women with normoglycemia after gestational diabetes showed lower insulin secretion for a given insulin resistance and lower hepatic insulin clearance than Nordic women [53].

### 3.2 Primary Hyperinsulinemia Theory

A differing perspective, the “primary hyperinsulinemia theory,” proposes that hyperinsulinemia, rather than being a consequence of obesity or T2D, is indeed a driver in their development [54, 55, 56, 57] (Figure S3). Hyperinsulinemia can be evident long before the development of impaired glucose regulation/obesity [58] and is an independent predictor of T2D; indeed, in healthy normoglycemic individuals, fasting hyperinsulinemia is predictive of T2D over 24 years of follow‐up [59].

Hyperinsulinemia may contribute to the development of obesity considering its physiological properties as an anabolic hormone acting on multiple tissues. Insulin causes expansion of fat mass by increasing lipogenesis and impairing lipolysis in adipose tissue. Insulin is also involved in appetite regulation, controlling satiety and controlling energy expenditure through its effects in the brain, most notably in hypothalamic nuclei. Hyperinsulinemia may also drive T2D occurring upstream of, and by inducing, insulin resistance.

There are multiple convincing lines of evidence to suggest that hyperinsulinemia, particularly in the basal state, is a primary defect that contributes to or helps maintain insulin resistance. This contrasts with the more prevalent view, articulated above, that instead insulin resistance represents the dominant and primary pathophysiological defect in T2D. One piece of supporting evidence is that rodents transfected with additional copies of the insulin gene, despite being normal weight, are insulin resistant with higher basal plasma insulin concentrations and hyperglycemia [54]. Similarly, patients with insulinoma, with elevated basal levels of insulin, are insulin resistant and have insulin (post‐receptor) signal transduction defects and impaired glucose‐induced insulin secretion. A range of animal models have shown reduction of circulating insulin by genetic engineering, and human studies with pharmacological suppression of insulin secretion with diazoxide or octreotide are associated with reduced adiposity/fat mass [60]. Prolonged exposure to hyperinsulinemia may lead to insulin resistance and hyperglycemia by insulin receptor desensitization and removal from the plasma membrane [61, 62].

### 3.3 Hyperinsulinemia in African Caribbeans

Compared to their White European counterparts, both normal‐weight and Black African women living with obesity demonstrate significantly lower insulin sensitivity, even considering equivalent body fat percentage and fasting glycemia. However, a characteristic feature is their tendency towards hyperinsulinemia, occurring to a greater extent than would be expected based on differences in insulin sensitivity [63, 64]. These observations imply that for any given degree of insulin sensitivity, either that first‐phase insulin secretion is greater or that hepatic insulin clearance is lower among Black Americans than White [65, 66, 67].

Lack of awareness of ethnic differences in hepatic insulin clearance may lead to misinterpretation of the measures, widely used, for insulin resistance, such as OGTT‐based measures and HOMA‐IR. The HOMA‐IR has been validated as a marker of insulin sensitivity in White European populations but has shown poor correlation with insulin sensitivity assessed by the Frequently Sampled Intravenous Glucose Tolerance Test (FSIVGTT) or hyperinsulinemic clamp in Jamaican adults without diabetes [68].

### 3.4 Hyperinsulinemia Is Also Implicated in Pathogenesis of Hypertension in Black Africans

Hyperinsulinemia has also been implicated in the higher risk of hypertension and suffering haemorrhagic stroke in African Caribbeans. Among African Caribbeans, there was a consistent relationship of insulin resistance with blood pressure, present from youth and remaining significant even with adjustment for adiposity [69]. Insulin has an anti‐natriuretic action by increasing sodium reabsorption in multiple nephron segments, via activation of IRS‐2 leading to expansion of blood volume. Hyperinsulinemia can also stimulate the sympathetic nervous system, which may stimulate renin secretion and increase peripheral resistance and hypertension [70].

In summary, hyperinsulinemia may not be a defect secondary to peripheral insulin resistance but in Black Africans may represent a primary defect with hyperinsulinemia related to impaired hepatic insulin clearance. Secondary insulin resistance may follow. This pathophysiological sequence (hyperinsulinemia and secondary insulin resistance) seems unique to Black Africans.

## 4 Lipid Metabolism

### 4.1 Adipose Tissue and Regulation of Fat Metabolism Under Normal Conditions

Lipid metabolism considers plasma fatty acids, not esterified to glycerol (NEFAs), as the central carriers of energy with TG as a storage form, hydrolyzed to release NEFAs according to metabolic demand. Adipose tissue, skeletal muscle, and the liver represent the three most quantitatively important tissues for lipid metabolism, each with their own TG depot. In adipose tissue, TGs can be released into the systemic circulation for delivery to other tissues, in skeletal muscle used as a substrate for oxidation, and in the liver can be re‐esterified to make TG or secreted in very low‐density lipoprotein (VLDL).

The principal function of adipose tissue is to ensure efficient and metabolically “safe” storage of post‐prandial dietary fat, buffering influx of dietary fat into adipose tissue storage, but mobilizing TG as an energy substrate in the post‐absorptive state [71]. Ineffective storage of dietary fat has been demonstrated in men living with abdominal obesity and implicated in the accumulation of ectopic fat [72].

**FIGURE 3:**
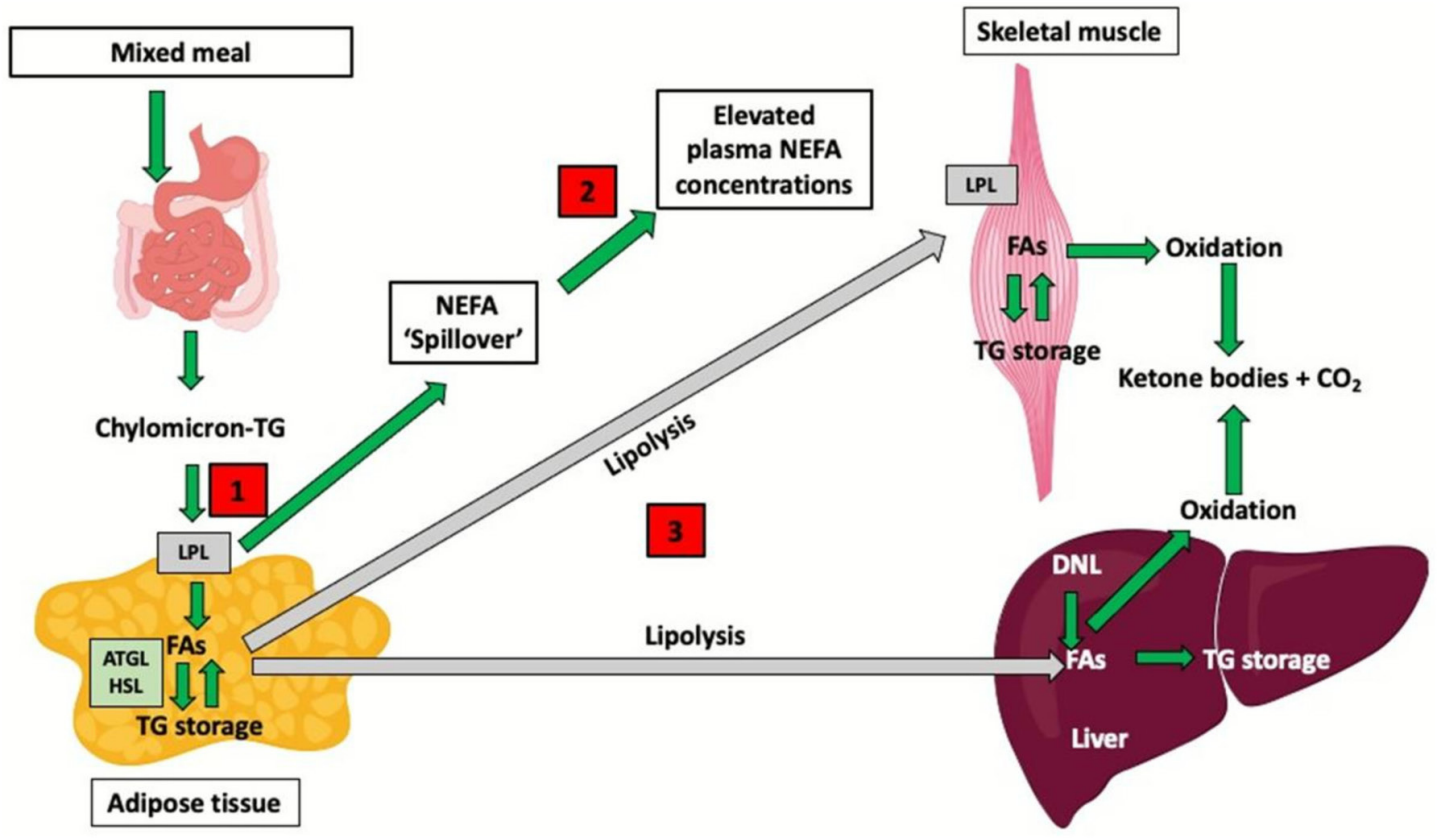
Potential sites of lipid dysregulation contributing to ethnic differences in cardiometabolic disease: (1) Impaired LPL‐mediated hydrolysis of chylomicron‐TG, leading to postprandial lipemia; LPL, in the capillary endothelium of adipose tissue and skeletal muscle, hydrolyzes TG‐rich lipoproteins (both chylomicrons and VLDL) to produce NEFAs for subsequent uptake. LPL is rate limiting for the vascular removal of chylomicron‐ and VLDL‐TG. LPL acts as a “metabolic gatekeeper,” central to energy partitioning, by virtue of its *tissue‐specific regulation and dependent on the physiological circumstances*. After feeding, insulin stimulates adipose tissue LPL activity (promoting fat storage) while inhibiting LPL activity in skeletal muscle [73]. In contrast, exercise stimulates LPL in skeletal muscle (promoting oxidation of NEFAs for fuel) with minimal effect on adipose tissue activity [74]. (2) Inadequate sequestration of meal fatty acids (FAs) into subcutaneous adipose tissue; if LPL activity is impaired, and NEFAs are not sequestered into adipose tissue, *spillover* into the systemic circulation can occur. Extra‐adipose tissues, including the liver, skeletal muscle, and pancreatic beta cell, are exposed to excessive fluxes of lipid fuels, TG accumulation driving lipotoxcity, insulin resistance and impaired insulin secretion. Although *spillover* occurs in healthy individuals [75], it has been associated with poor metabolic health. In a long‐term overfeeding study, with significant weight gain, increased NEFA *spillover* was demonstrated after a test meal, and the magnitude of *spillover* correlated closely with the overfeeding‐induced change in visceral adipose tissue (VAT) [76]. (3) Elevated subcutaneous adipose tissue lipolysis, the main determinant of plasma non‐esterified fatty acid (NEFA) concentrations; this is partly regulated by ATGL and HSL, which are inhibited by insulin but stimulated under fasting conditions. Abnormal insulin‐mediated suppression of plasma NEFA has been shown to be an early defect in the development of T2D. Insulin resistant individuals have been shown to have lower adipose tissue fatty acid sequestration postprandially, despite the presence of hyperinsulinemia [72]. NEFA efflux from enlarged and lipolytically active visceral fat depots are thought by some to play a major role in the elevation of plasma NEFA concentrations. These NEFAs enter the portal vein directly, increasing FA delivery to the liver. ATGL, adipose triglyceride lipase; DNL, de novo lipogenesis; HSL, hormone‐sensitive lipase.

Fatty acid trafficking, with efficient switching between *TG mobilization* (lipolysis of adipose tissue TG to mobilize NEFAs in the post‐absorptive state) and *storage* (re‐esterification in adipose tissue, liver, and other tissues during the postprandial state), occurs in cycles according to nutritional state. Normal fatty acid trafficking plays a prominent role in the maintenance of metabolic health, principally regulated through the intracellular lipases, hormone‐sensitive lipase (HSL), adipose tissue triglyceride lipase (ATGL), and the extracellular lipase, lipoprotein lipase (LPL). Insulin regulates LPL and HSL activity, stimulating LPL and inhibiting HSL in adipose tissue. There are three key metabolic steps in fatty acid trafficking, each a potential site of dysregulation, shown in Figure 3.

The liver is a central organ for lipid metabolism. NEFAs may accumulate from the systemic circulation or be produced by de novo lipogenesis (DNL). Hepatic TG may be stored or exported in VLDL to provide TG to other tissues. VLDL production and secretion are inhibited. In insulin resistance, loss of this inhibition results in VLDL hypersecretion.

### 4.2 Ethnic Differences in Lipid Metabolism

TGs and NEFAs are present in the circulation in varying quantities dependent on dietary intake, body composition, insulin resistance, and glucose tolerance. Differences in fat metabolism are reflected in the post‐absorptive (fasting) and/or postprandial concentrations of TGs and NEFAs.

### 4.3 TGs

#### 4.3.1 Fasting and Postprandial Plasma TG Concentrations in Africans

Fasting TGs are significantly lower in normoglycemic and glucose intolerant African Caribbean men and in normoglycemic African Caribbean women than in White European men/women [77]. A more recent study of African Caribbean and White European men with normal glucose tolerance, impaired glucose tolerance, and early T2D showed that when the groups were combined, fasting TGs were lower in African Caribbean men [78]. In the T2D group, although TGs were lower, this was not statistically significantly different, whereas in subjects with normal glucose tolerance, TGs were significantly lower [79].

In the United States, plasma TGs are lower in young, healthy African American men and women than in White American men and women, respectively [80]. This lower plasma TG in African Americans is apparent at a young age because African American adolescents (mean age 14.7 ± 2.9, combined male and female) also have lower TG and total and large VLDL compared to White American adolescents [81]. Young people aged 8–17 years have lower plasma TG and smaller VLDL size in both male and female African Americans compared to White Americans [82].

The mechanism for lower fasting TG in African Caribbean people may be reduced hepatic production of VLDL and/or greater peripheral tissue clearance. Plasma TG, VLDL‐TG, and VLDL‐TG production rate has been shown to be lower in African American women than White women, but VLDL‐TG clearance was not different [83]. The reduced VLDL‐TG production rate was due to a lower contribution from nonsystemic sources of NEFAs (DNL and intrahepatic fat and visceral adipose). The molar ratio of VLDL‐TG and VLDL apoB‐100 was also lower in African American women, demonstrating they produced smaller VLDL with less TG [84]. No studies have measured fasting VLDL kinetics in African American or African Caribbean men.

Fasting large, medium, and small triglyceride‐rich lipoprotein concentrations (TRLP) have also been shown to be lower in pre‐ and postmenopausal African American women than White American women [85]. However, very small TRLP were not significantly different; these particles likely represent IDL and remnant chylomicrons, contain up to 33% of non‐fasting cholesterol, and are highly atherogenic.

After a high‐fat meal, postprandial TGs are lower in lean African American women than White women [86]. In lean African American men, the incremental (above basal) change in TG following a fat meal was significantly lower than White men at 2 h and tended to be lower at 3 and 4 h after the fat load [87]. The lower postprandial TGs may be due to more efficient clearance of chylomicrons and VLDL, perhaps explained by higher post‐heparin LPL activity in African American men [84, 87]. TGs have been shown to be inversely related to LPL activity in both African American and White American [87]. Higher insulin levels in African American [88] people may also contribute to greater clearance of TGs in African American because insulin activates LPL in vivo [89].

#### 4.3.2 Fasting and Postprandial Plasma TG Concentrations in South Asians

Unlike people of African ancestry, fasting plasma TGs in South Asians have been reported to be either higher [84, 88] or not different from men or women of European origin [86, 87]. In the studies that found higher TGs in South Asian men, subjects were well matched for weight and BMI, but percentage body fat was considerably higher than in White Europeans. When corrected for this, the difference in plasma TGs was not significant in one study [90]. In the NHANES and Center for Cardiometabolic Risk Reduction in South Asia, although waist circumference and mean BMI were lower in the South Asians, plasma TGs were greater than in White Americans (a combined group of men and women) [91].

The mechanism for higher TGs in some studies is far from clear. There have been no measurements of VLDL production rate in South Asians. However, a US study of hepatic DNL in 15 South Asians and 15 White American young healthy males and females matched for BMI demonstrated a higher hepatic DNL in South Asians in response to a high sugar drink (1.5 g/kg fructose + 1.5 g/kg glucose), compared to White Americans matched for BMI. Although fasting plasma TGs were not different, the increase in plasma TGs and VLDL TGs 4 h after the drink was higher in the South Asians. A higher hepatic DNL could contribute to the increased liver TG reported in South Asians [92] and the higher plasma TGs reported in some studies.

A small study of postprandial TGs in South Asians (*n* = 8) and White European (*n* = 9) men and women matched for BMI found no difference between ethnic groups [93]. However, two studies found postprandial TGs were higher in South Asian men than White European men [90, 94]. In both these studies, the difference in postprandial TGs may be due to differences in body fat rather than ethnicity. More convincing evidence for higher postprandial TGs in South Asians comes from a study that found higher postprandial TGs in lean premenopausal South Asian versus White European women and in South Asian men living with central obesity than in White European men. The women had similar BMI, waist circumference, and body fat, and the men were selected by ethnic‐specific waist circumference cut‐points [74].

### 4.4 NEFAs

#### 4.4.1 Plasma NEFAs in African People

Although there are distinct differences in TGs between people of African Caribbean and White European ancestry, the differences in NEFAs are less clear. No difference was found in fasting NEFAs between healthy middle‐aged White European and African Caribbean men matched for BMI and fat mass [95]. There was also no difference in NEFA flux and concentration measured with an infusion of U^13^C palmitate in young, healthy African American and White American men and women well matched for BMI (mean BMI 27 kg/m^2^) [80]. However, differences were revealed when subjects with obesity were studied. Fasting plasma NEFA concentration and production rate were lower in African American women with obesity and T2D than in matched White American women [83].

Using an insulin‐modified FSIVGT and minimal model for NEFA, insulin‐induced suppression of NEFAs was greater in premenopausal African American than White American women (BMI 30 kg/m^2^) [88]. This could be driven by the higher insulin concentrations in African Americans, inhibiting HSL. In subcutaneous and omental adipose tissue biopsies, HSL mass and basal lipolytic rates were lower in African American women with obesity compared to White American [96]. Further evidence for lower lipolysis in African Americans comes from the measurement of in vivo TG flux using ^2^H_2_O in adipose tissue in African American women with obesity. TG synthesis (and breakdown) was lower than in matched White American women [97].

Although some studies suggest lower fasting lipolysis in men and women of African ancestry, feeding studies reveal a very different picture. After a meal high in glucose, postprandial NEFAs were higher in borderline overweight African Caribbean men than in White European men [98] with no difference in insulin response between the two groups. NEFAs were also higher, 30 min after a mixed meal in pre‐ and postmenopausal African American women despite a greater insulin response than White women [99]. The difference between ethnicities was more pronounced in the postmenopausal women.

#### 4.4.2 Plasma NEFAs in South Asian People

Very few studies have measured fasting NEFAs in South Asians. Zoratti et al. reported no difference in fasting plasma NEFA in South Asian men compared to White European men matched for BMI and fat mass. Hall et al. also reported no difference in fasting NEFAs in South Asian versus White European men matched for BMI [100].

There was also no difference in postprandial NEFAs in lean premenopausal women compared with WE women or South Asian men with central obesity compared with WE men [101]. Only one study has reported a difference in NEFAs in this ethnic group. Insulin suppression of NEFAs during a clamp in South Asian and Norwegian men and women with T2D found reduced suppression in the South Asians indicating adipose tissue insulin resistance despite considerably lower BMI (31 vs. 37 kg/m^2^) and total fat mass (28 vs. 40 kg) in the South Asians [102]. Because HbA1c was significantly higher in the South Asians, poorer glycemic control may account for the differences.

In summary, there are clear‐cut differences in lipid metabolism in Africans, but the differences are less apparent in South Asians compared to White Europeans. There have been very few studies of plasma TG and NEFA flux, and considerable more research is needed to unravel the mechanism for the differences in concentration reported in some studies. This may lead to a better understanding of the mechanism by which fat deposition occurs into ectopic fat depots.

### 4.5 Summary

**FIGURE 4:**
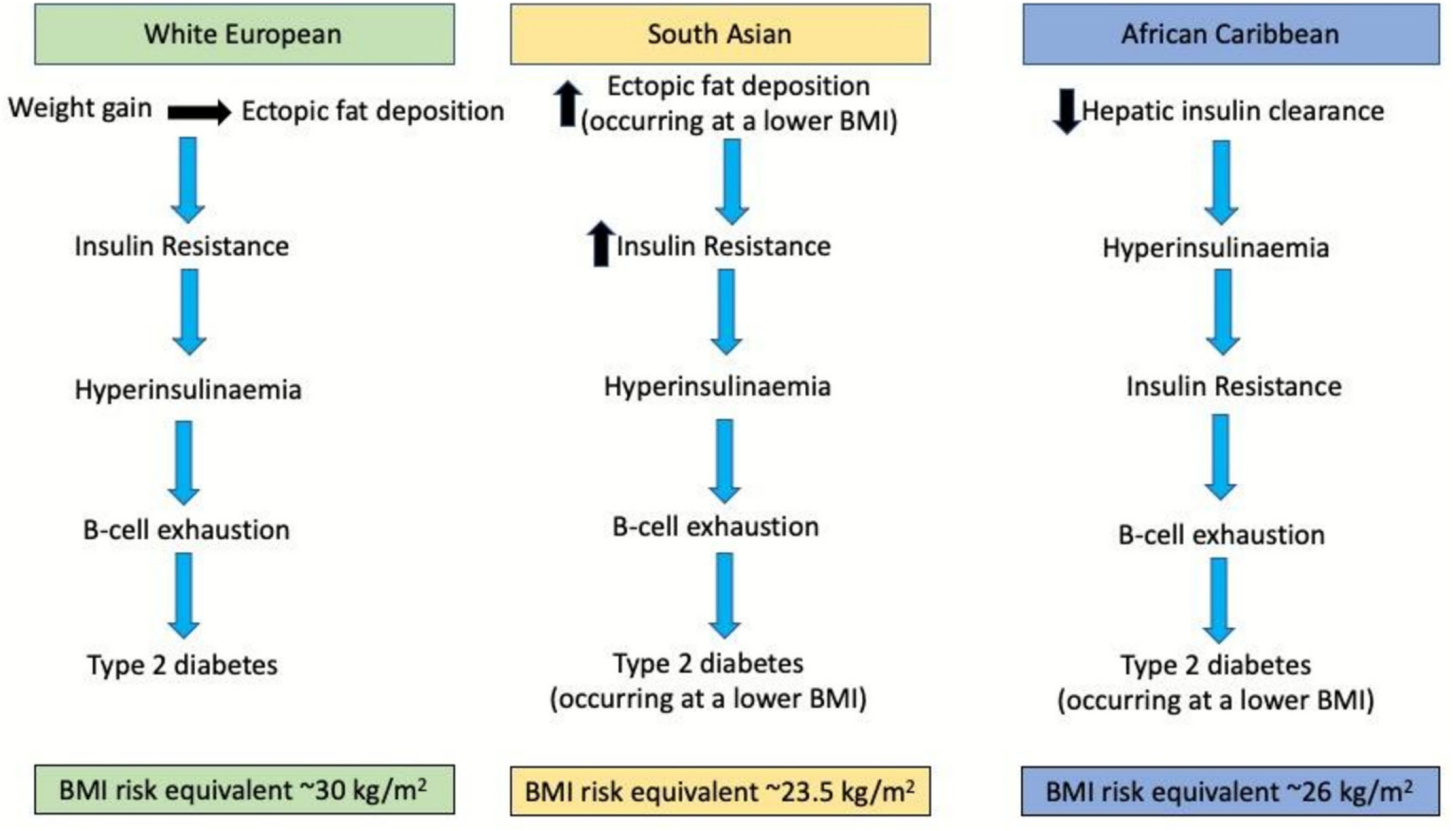
Possible pathophysiological defects and their temporal associations in people of White European, South Asian, and African Caribbean ancestry.

Amassing all the evidence from above, there are complex considerations when trying to determine the mechanistic basis for the differential susceptibility to obesity‐related complications such as T2D in different ethnic minority groups. Aside from genetic, socioeconomic, cultural, political, behavioral, and psychological factors and considering only the biological basis for this phenomenon, the explanation is likely multifactorial and complex. Based on the physiological evidence, it would seem likely that different pathophysiological factors contribute to specific ethnic populations (Table 1 and Figure 4). Parallel mechanistic studies in well‐matched ethnic populations utilizing metabolic assessments and multiorgan imaging can untangle the complex pathophysiology of T2D and MASLD across different ethnic groups.

**TABLE 1:** Ethnic‐specific differences in body composition, pathophysiological drivers of cardiometabolic disease, and disease associations in people of White European, South Asian, and African Caribbean ancestry. The White European represents the reference group.

	White Europeans	South Asians	African Caribbean
Biochemical changes (TGs and NEFAs)			
Fasting TGs	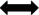	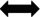 OR 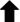	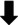
Postprandial TGs	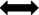	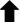	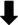
Fasting NEFAs	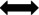	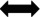	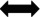 OR 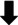
Postprandial NEFAs	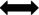	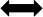	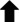
Body composition			
Subcutaneous adipose tissue (SAT) volume	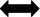		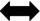
Visceral adipose tissue (VAT) volume	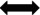	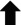	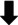
SAT/VAT ratio	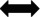	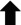	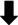
Liver fat	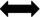	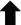	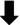
Pancreatic fat	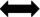	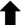	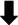
Muscle mass	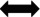	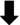	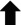
Pathophysiological drivers			
Insulin resistance	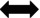	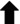	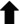
Insulin secretion	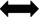	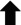	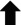
Insulin clearance	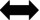	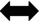	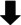
Disease associations			
Prevalence of obesity	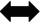	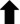	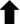
Prevalence of type 2 diabetes	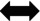	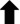	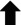
Prevalence of MASLD	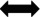	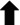	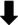
Prevalence of CVD	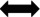	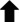	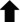

## Funding

The authors have nothing to report.

## Conflicts of Interest

D.J.C. has received investigator‐initiated grants from AstraZeneca and Novo Nordisk and support for education from Perspectum with any financial remuneration from pharmaceutical company consultation made to the University of Liverpool. U.A. has received honoraria from Procter & Gamble, Viatris, Grunenthal, and Sanofi for educational meetings and funding for attendance to an educational meeting from Diiachi Sankyo. U.A. has also received investigator‐led funding by Procter & Gamble and is a council member of the Royal Society of Medicine's Vascular, Lipid & Metabolic Medicine Section. M.W. has received consulting fees from Novo Nordisk, as well as investigator‐initiated grants for various related projects. The other authors declare no conflicts of interest.

## Supporting information


**Figure S1:** Traditional model of Type 2 diabetes with insulin resistance (IR) as the primary defect with secondary hyperinsulinemia.
**Figure S2:** South Asian model of Type 2 diabetes with greater ectopic fat and thus more profound insulin resistance (IR) as the primary defect with secondary hyperinsulinemia.
**Figure S3:** Black African model of Type 2 diabetes with insulin resistance (IR) as the primary defect with secondary insulin resistance.

## Data Availability

Data sharing is not applicable to this article as no new data were created or analyzed in this study.
