# Supplementary material for: Differential Pathophysiological Drivers of Susceptibility to Type 2 Diabetes and Metabolic Dysfunction–Associated Steatotic Liver Disease: Ethnic Differences in Insulin Dynamics, Whole‐Body Fat Metabolism, and Organ‐Specific Lipid Deposition
Source: Obes Rev. 2026 Feb 16;27(8):e70104. doi: 10.1111/obr.70104 (PMC13371816; doi:10.1111/obr.70104)
Supplement: Supplementary file 1 — Figure S1: Traditional model of Type 2 diabetes with insulin resistance (IR) as the primary defect with secondary hyperinsulinemia. Figure S2: South Asian model of Type 2 diabetes with greater ectopic fat and thus more profound insulin resistance (IR) as the primary defect with secondary hyperinsulinemia. Figure S3: Black African model of Type 2 diabetes with insulin resistance (IR) as the primary defect with secondary insulin resistance. [file OBR-27-e70104-s001.pdf]

**Supplementary Material**

Differential pathophysiological drivers of susceptibility to type 2 diabetes and metabolic dysfunction-associated steatotic liver disease: ethnic differences in insulin dynamics,  whole body fat metabolism and organ-specific lipid deposition.

Daniel J Cuthbertson[1,2], Martin Whyte[3,4], Alex E Henney[1,2], Uazman Alam[1,2], Louise Goff[5,6], Barbara A Fielding[3], A. Margot Umpleby[3]

1Department of Cardiovascular and Metabolic Medicine, Institute of Life Course and Medical Sciences, University of Liverpool, Liverpool, UK

2University Hospital Aintree, Liverpool University Hospitals NHS Foundation Trust, Liverpool, UK

3Department of Nutrition, Food and Exercise Sciences, Faculty of Health and Medical Sciences, University of Surrey

4King's College Hospital, London

5King's College London,

6Leicester Diabetes Centre, Leicester General Hospital and University of Leicester, Leicester.

**Corresponding author**

Dr Alex E Henney

University of Liverpool, Department of Cardiovascular and Metabolic Medicine

Ahenney@liverpool.ac.uk

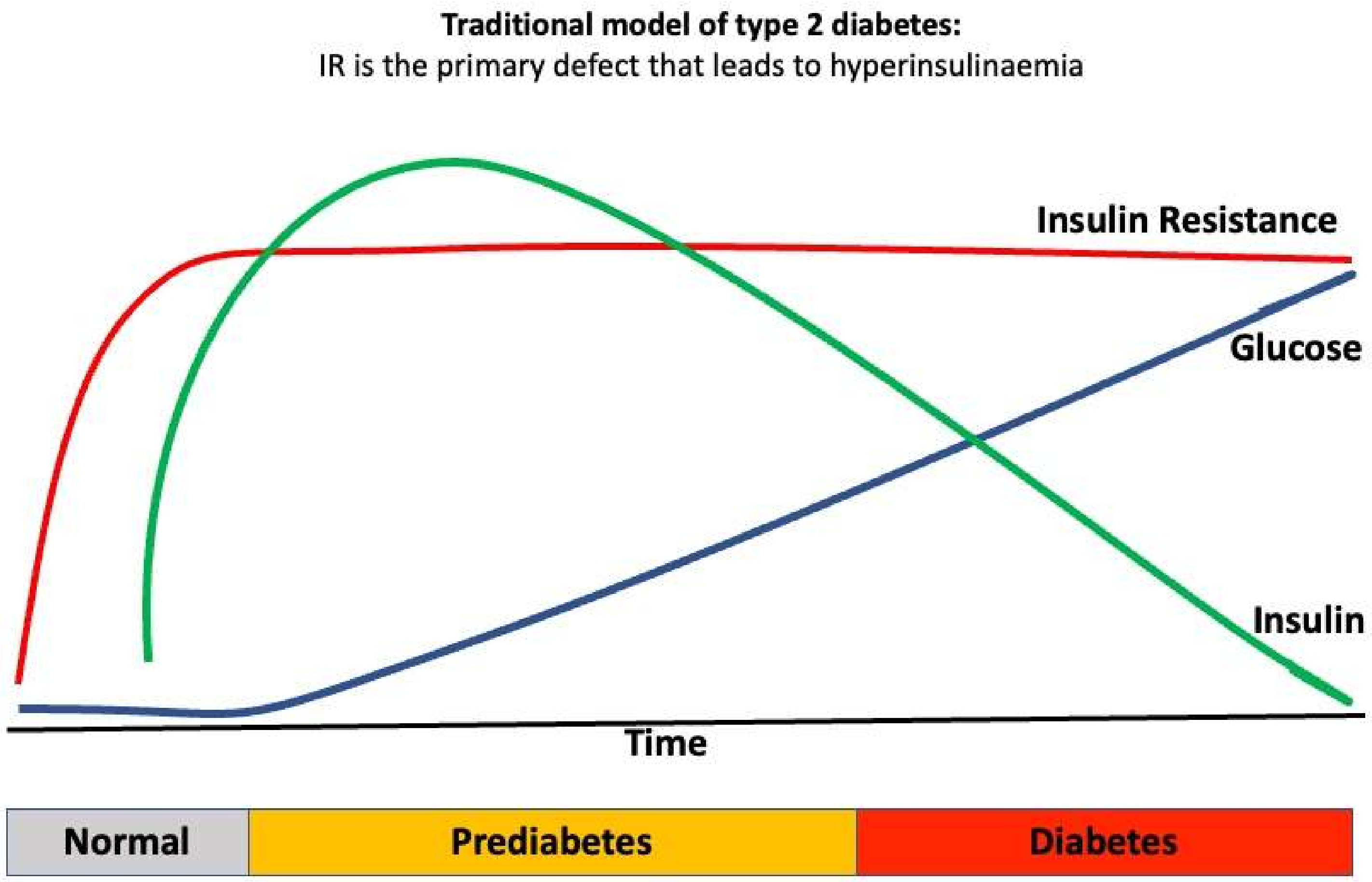


**Supplementary Figure 1** Traditional model of type 2 diabetes with insulin resistance (IR) as the primary defect with secondary hyperinsulinaemia

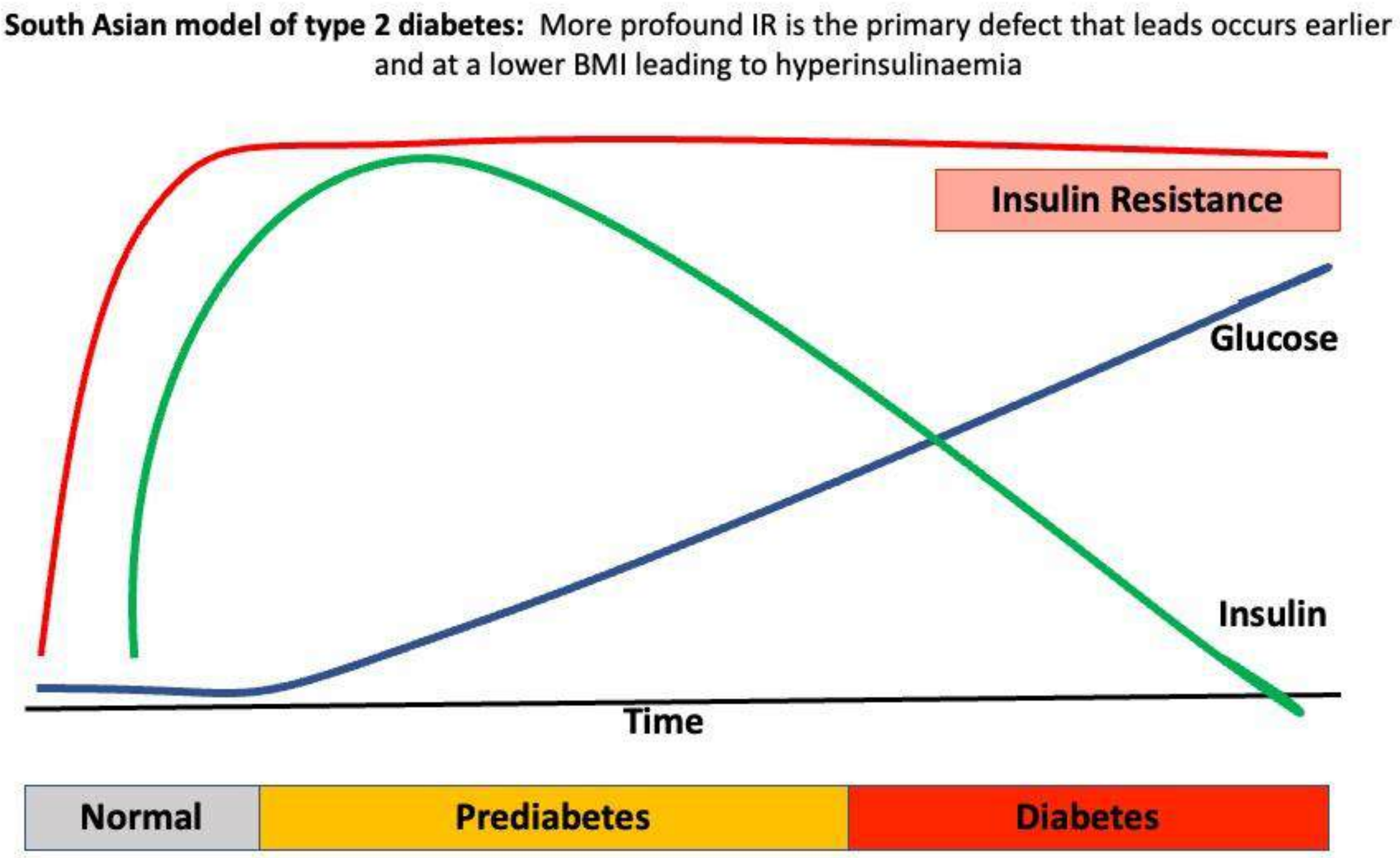


**Supplementary Figure 2** South Asian model of type 2 diabetes with greater ectopic fat and thus more profound insulin resistance (IR) as the primary defect with secondary hyperinsulinaemia

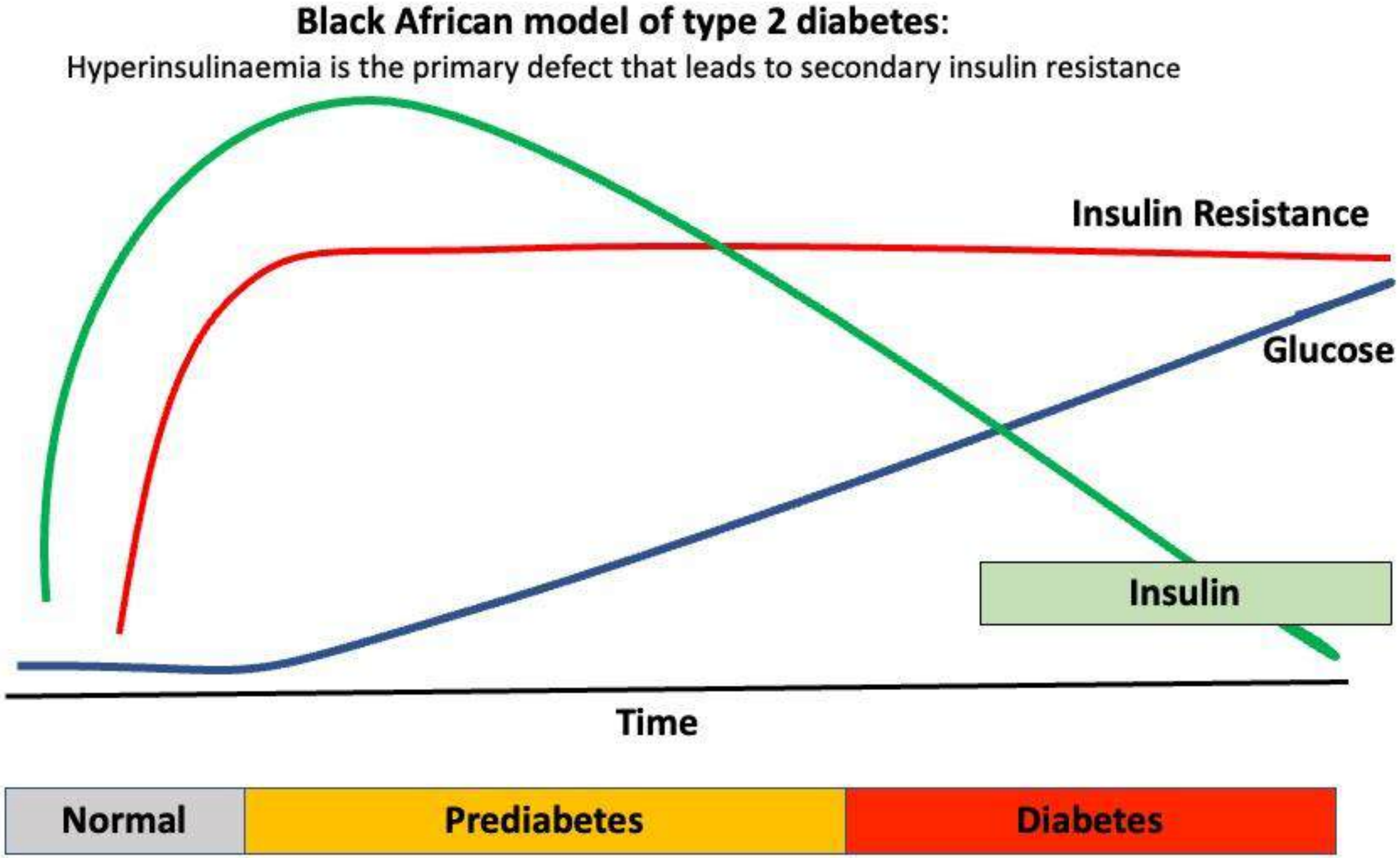


**Supplementary Figure 3** Black African model of type 2 diabetes with insulin resistance (IR) as the primary defect with secondary insulin resistance
